# Integrating Multi-Omics Data to Identify Key Functional Variants Affecting Feed Efficiency in Large White Boars

**DOI:** 10.3390/genes15080980

**Published:** 2024-07-25

**Authors:** Yue Xiang, Jiahui Sun, Guojian Ma, Xueting Dai, Yuan Meng, Chong Fu, Yan Zhang, Qiulin Zhao, Jingjin Li, Saixian Zhang, Zhuqing Zheng, Xinyun Li, Liangliang Fu, Kui Li, Xiaolong Qi

**Affiliations:** 1 Guangdong Laboratory of Lingnan Modern Agriculture, Key Laboratory of Livestock and Poultry Multi-Omics of MARA, Agricultural Genomics Institute at Shenzhen, Chinese Academy of Agricultural Sciences, Shenzhen 518000, China; yxiang@webmail.hzau.edu.cn (Y.X.); mengyuan@caas.cn (Y.M.); jingjinli@webmail.hzau.edu.cn (J.L.); zhangsaixian@webmail.hzau.edu.cn (S.Z.); likui@caas.cn (K.L.); 2 Key Lab of Agricultural Animal Genetics, Breeding and Reproduction of Ministry of Education and Key Laboratory of Swine Genetics and Breeding of Ministry of Agriculture, College of Animal Science and Technology, Huazhong Agricultural University, Wuhan 430070, China; sun_jiahui314@webmail.hzau.edu.cn (J.S.); maguojiancofco@163.com (G.M.); daixueting@webmail.hzau.ed (X.D.); fuchong@webmail.hzau.edu.cn (C.F.); zhangy2114@webmail.hzau.edu.cn (Y.Z.); qiulinzhao@webmail.hzau.edu.cn (Q.Z.); zzq_1207@mail.hzau.edu.cn (Z.Z.); xyli@mail.hzau.edu.cn (X.L.); fuliangliang2017@mail.hzau.edu.cn (L.F.)

**Keywords:** pigs, feed efficiency, ATAC-seq, GWAS, duodenum, muscle

## Abstract

**Abstract:** Optimizing feed efficiency through the feed conversion ratio (FCR) is paramount for economic viability and sustainability. In this study, we integrated RNA-seq, ATAC-seq, and genome-wide association study (GWAS) data to investigate key functional variants associated with feed efficiency in pigs. Identification of differentially expressed genes in the duodenal and muscle tissues of low- and high-FCR pigs revealed that pathways related to digestion of dietary carbohydrate are responsible for differences in feed efficiency between individuals. Differential open chromatin regions identified by ATAC-seq were linked to genes involved in glycolytic and fatty acid processes. GWAS identified 211 significant single-nucleotide polymorphisms associated with feed efficiency traits, with candidate genes *PPP1R14C*, *TH*, and *CTSD*. Integration of duodenal ATAC-seq data and GWAS data identified six key functional variants, particularly in the 1500985–1509676 region on chromosome 2. In those regions, *CTSD* was found to be highly expressed in the duodenal tissues of pigs with a high feed conversion ratio, suggesting its role as a potential target gene. Overall, the integration of multi-omics data provided insights into the genetic basis of feed efficiency, offering valuable information for breeding more efficient pig breeds.

## 1. Introduction

In recent years, the availability of food for both humans and animals has become increasingly limited because of continuous population growth and a reduction in arable land area worldwide [1]. Thus, there is increasing awareness of the importance of maximizing the efficiency of livestock production. One approach is to increase the efficiency with which animals convert feed into meat and other products, such as eggs or milk. A commonly used metric for measuring this trait, called feed efficiency, is the feed conversion ratio (FCR), which is calculated as the ratio of feed intake to weight gain [2,3]. The FCR is also positively correlated with average daily gain [4].

Feed is a source of energy and nutrients necessary for fulfilling essential functions such as muscle growth and fat deposition. In pigs, FCR is intricately linked to digestion, the absorption of nutrients in the small intestine, and energy metabolism in muscle tissues [5]. For example, expression levels of some genes are significantly correlated with FCR. For instance, the expression levels of *IGF1*, *IGF2*, *FGF2*, and *FGF7* (insulin-like growth factor) in the intestine are closely linked to growth rate and feed conversion efficiency in pigs [6]. Additionally, certain transcription factors such as PPARα, PPARγ, and RXRα are expressed in the intestinal epithelium, where they regulate fatty acid metabolism and absorption, thereby influencing the energy conversion efficiency in the animal’s gut [7,8]. Increasing research suggests that the duodenum, the initial stage of nutrient absorption in the small intestine, plays a crucial role in maintaining the digestive environment and the body’s nutritional balance. Additionally, it can regulate pancreatic and liver functions, thereby influencing energy metabolism [9,10]. Furthermore, energy metabolism in the muscle and the growth and development of muscle tissue are also closely associated with feed efficiency [11].

Whole-genome association studies (GWASs) have identified variant loci or candidate genes related to feed efficiency traits in pigs, providing insights into the genetic architecture of complex traits [12,13,14,15,16]. However, the majority of reported genetic variant loci are currently situated in non-coding regions, posing challenges in thoroughly investigating the mechanisms behind phenotypic changes influenced by single-nucleotide polymorphisms (SNPs) within these regions, thereby hindering the identification of causal variants associated with the phenotype. One approach to meet this challenge is to use multi-omics technologies to reveal the regulatory mechanisms of gene expression. This knowledge is crucial for deciphering the associations between genes identified in GWASs and the corresponding traits.

In this study, we performed an integrative multi-omics analysis, utilizing genotype imputation for a GWAS to combine genetic variations with epigenetic and transcriptomic data, aiming to comprehend the relationship between genetic variations and phenotypes at different levels. We used the assay for transposase-accessible chromatin with sequencing (ATAC-seq) to identify differential open chromatin regions (OCRs) and RNA sequencing (RNA-seq) to identify differentially expressed genes (DEGs) in the duodenum and muscle tissues of high- and low-feed-efficiency pigs. Subsequently, we dissected 211 GWAS signals associated with feed efficiency traits, identifying key functional variants influencing pig feed efficiency.

## 2. Materials and Methods

### 2.1. Collection of Muscle and Duodenal Tissue Samples

In this study, we utilized purebred large white boars, three with extremely high FCR values and three with extremely low FCR values. These pigs were raised on the same commercial farm (Wuhan COFCO Meat Products Co. Wuhan, China) under similar conditions and fed the same diet. We ensured that all experimental animals were kept under identical rearing conditions, including the same feeding regimen and growth environment. We strictly controlled all environmental variables, such as temperature, humidity, and lighting, to minimize their impact on the experimental results. Additionally, the selection of experimental animals came from a large population to ensure the reproducibility and representativeness of the experiment. Thus, we can assure that the performance differences among these animals were primarily due to genetic reasons rather than external factors such as pathogens or stress. All pigs were euthanized at 180 days of age after an overnight fasting period (HZAUSW-2023-0023). The euthanasia process involved a mixture of azaperone (4 mg/kg) and atropine (0.04 mg/kg) administered via intramuscular injection into the muscle-rich area of the neck’s dorsal side to sedate the pigs. After 10 min, once the pigs were sedated, a mixture of xylazine (4 mg/kg) and Zoletil (2 mg/kg) was injected intramuscularly into the neck muscles to anesthetize them. Longissimus dorsi and duodenal samples were collected and stored at −80 °C for subsequent experiments. The sampling procedures received approval from the Ethics Committee of Huazhong Agricultural University. RNA-seq analysis was conducted on samples from three pigs with the highest FCR levels and three with the lowest FCR levels. In addition, ATAC-seq analysis was performed on samples from two pigs with extremely high FCR levels and two with extremely low FCR levels.

### 2.2. Construction of RNA-Seq Libraries

The muscle and duodenal tissues were pulverized into powder by grinding in liquid nitrogen. Approximately 60 mg of muscle tissue was used for RNA-seq library construction. TRIzol solution (1 mL) was added to a centrifuge tube containing either muscle or duodenal tissue for tissue lysis. RNA extraction was then performed following the protocol provided by the manufacturer (Thermo Fisher Scientific, Waltnam, MA, USA). The extracted RNA was quantified using a Qubit fluorometer. RNA enrichment was performed using VAHTS mRNA Capture Beads (Vazyme, Nanjing, China). Subsequently, library construction for RNA-seq was carried out following the protocol provided by the VAHTS Universal V8 RNA-seq Library Prep Kit for Illumina (Vazyme, Nanjing, China).

### 2.3. Construction of ATAC-Seq Libraries

Muscle and duodenal tissues were pulverized into powder by grinding in liquid nitrogen. Approximately 20 mg of tissue was used for ATAC-seq library construction. The powdered tissue was resuspended in 1 mL of nuclei extraction buffer (Miltenyi Biotec 130-128-024) and incubated on ice for cell lysis (12 min for muscle tissue and 10 min for duodenal tissue). The sample was centrifuged at 1000× *g* for 5 min at 4 °C to collect the pellet. The pellet was resuspended in 1 mL of DPBS and filtered through a 70 μm cell strainer (FALCON, Corning, NY, USA) to obtain nuclei. Nuclei were counted using a hemocytometer, and 50,000 nuclei were used for subsequent experiments. The nuclei were resuspended in a 50 μL tagmentation system (1 μL Tn5, 10 μL 5× TAG, 0.5 μL 10% Tween-20, 38.5 μL nuclease-free ddH_2_O) and incubated in a ThermoMixer at 37 °C at 1000 rpm for 1 h. A Zymo Research DNA Clean & Concentrator 5 kit was used to recover DNA fragments. The quality control and library preparation were performed as previously described [17]. The final ATAC-seq library was electrophoresed in a 2% agarose gel at 100 V for 1 h. After electrophoresis, DNA fragments in the 100–1000 bp range were excised from the gel. The purified and recovered DNA library was then subjected to sequencing on an Illumina NovaSeq6000 instrument (Illumina, San Diego, CA, USA).

### 2.4. Collection of Animal Population Data, Phenotypic Data, and Genotyping

These animals were kept under uniform feeding and management conditions during the measurement period and were housed in standard commercial pig barns. Throughout the experiment, feeding records were automatically generated by an automated feeding station (Pig Performance Testing System, Nedap, Groenlo, The Netherlands). The data consisted of individual IDs, breeds, birthdates, initial weights, start dates, daily feed intakes, weight gain during the testing period, final weights, feed consumption, and end dates. Pig ear tissue samples, approximately the size of soybeans, were cut into small pieces and placed into 1.5 mL centrifuge tubes. After digesting the proteins with Proteinase K, genomic DNA was extracted using a Tecan Freedom EVO NGS workstation and MagPure Tissue DNA KF Kit (MD5112-02). All DNA samples had a concentration of ≥40 ng/µL and a quantity of ≥1 µg. Following quality control by a commercial sequencing company, library construction and sequencing were performed. Target SNP genotyping was performed by sequencing.

### 2.5. Extreme Phenotype Selection in Population Subgroups

The data were statistically analyzed using SPSS 26.0 software. A simple linear regression model, y = a + bx, was employed to depict the growth curve of each pig, correlating weight (y) with age in days (x). Analysis of residuals led to the exclusion of 33 pigs with outlier data. For the remaining 484 pigs with reliable average daily gain and FCR measurements, preliminary statistical analyses were conducted, namely, the calculation of descriptive statistics (the mean, standard deviation, and maximum and minimum values) for each phenotypic trait. Preliminary statistical analysis, including the calculation of descriptive statistics such as mean, standard deviation, and maximum and minimum values, along with a normal distribution test, was conducted using SPSS 26.0 (Supplementary Figure S1). Normality tests were performed. Based on FCR values, the three pigs with the highest FCRs were grouped as the high-FCR group, while the three pigs with the lowest FCRs were grouped as the low-FCR group (Supplementary Figure S6 and Table S3).

### 2.6. RNA-Seq Data Analysis

RNA-seq data were analyzed following the workflow provided by ENCODE. FastQC (v0.11.9) software was used to assess the raw data quality, and Trim Galore (v1.18) was then used to remove adapters and low-quality reads, resulting in high-quality clean reads. These clean reads were aligned to the reference genome using STAR (2.5.1b) software to determine their quality. RSEM (1.2.31) software was employed to quantify the RNA expression levels for each sample. Analysis of DEGs was conducted using the R (3.6.2) software package DESeq2 (1.26.0), with DEGs identified based on the criteria: |log2(fold change)| ≥ 1 and *p* < 0.05. The analysis was performed with three biological replicates.

### 2.7. ATAC-Seq Data Analysis

The ATAC-seq data were analyzed following the workflow provided by ENCODE. For all datasets, FastQC was used for initial quality control processing of raw sequence reads, and adapters and low-quality sequences were removed to obtain high-quality clean reads. Burrows–Wheeler Aligner (BWA, version 0.7.12) software was used to align sequencing reads to the reference genome (*Sus scrofa* 11.1). SAM files were converted to BAM format using Samtools and utilized for peak calling. MACS2 (version 2.1.2) software was employed for peak detection to provide an overview of OCRs across the entire genome for each sample. The DESeq2 package in R was used to analyze differences in peaks between groups based on the following criteria: |log2(fold change)| ≥ 0.58 and *p* < 0.05. Two biological replicates were used. To identify transcription factors with potential regulatory roles in the OCRs, motif enrichment analysis of differential OCRs was conducted using the findMotifsGenome.pl script in HOMER, specifying a motif analysis fragment length of 200. The specific command used was: findMotifsGenome.pl sample.bed Sscrofa11.1 output –size 200.

### 2.8. Gene Function Annotation

For the functional analysis of DEGs and genes regulated by differential peaks, Ensembl Biomart was used to convert pig gene IDs to human gene IDs. Subsequently, these IDs were uploaded to Metascape (https://metascape.org/gp/index.html#/main/step1 accessed on 13 February 2024) to obtain GO biological process functional annotations.

### 2.9. Genotyping and Quality Control

Genomic DNA was extracted from all samples using the bead method, and genotyping was performed using a Pig 80K functional SNP genotyping chip. Following quality control standards, SNPs with a call rate below 90% and SNPs with a minor allele frequency below 0.05 were excluded from all samples genotyped with each chip. These quality control procedures were executed using PLINK 1.9.

### 2.10. Genotype Imputation and Accuracy Assessment

The reference panel used in this study was constructed from highly credible sites obtained from the genomic sequences of 288 pigs selected for genetic representativeness in earlier lab experiments. Beagle 5.1 was employed to impute the 80K SNP chip data to the sequence data, with the following command: Java -Xmx40g -jar ~/01.software/install/beagle.22Jul22.46e.jar

gt = merge.filter.vcf.gz out = merge.filter.imp iterations = 15 nthreads = 6

The imputation of 80K SNP chip data to sequence data was performed on data from the 3866 pigs using QUILT v1.0.0 software with parameters set to nGen = 100, nGibbsSamples = 10, and nCores = 4. To assess the accuracy of genotype imputation, four randomly selected Duroc pigs were subjected to high-depth sequencing. The concordance rate between imputed and sequenced genotype data was computed using bcftools.

### 2.11. GWAS

GWAS was conducted for the corrected 30–100 kg FCR trait using the GCTA MLMA-LOCO (mixed linear model association analysis using a dense genetic relationship matrix) method. Fixed effects included the top five principal components from principal component analysis (PC1–PC5), pen information, birth farm, birth year, and birth month. Gender was not included, as all subjects were male pigs. Bonferroni correction in R was used to determine the genome-wide significance threshold, and SNPs were filtered based on the criterion *p* < 4.55E−08. Manhattan plots and box plots were generated using python (3.7.0) software in the R environment for visualization purposes.

## 3. Results

### 3.1. Mapping Quantitative Trait Loci for Pig Feed Efficiency

**Figure 1:**
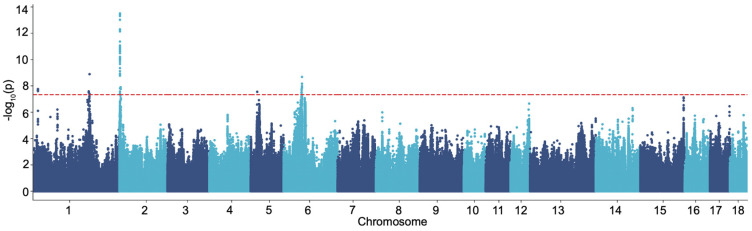
A Manhattan plot illustrating the relationship between all analyzed SNPs and FCR.

Genotype data for 3866 boars with recorded FCRs were obtained from an 80K functional SNP lipid chip (187,255 SNPs) and imputed to whole-genome sequence resolution. To evaluate the overall concordance between observed and imputed genotypes, four pigs were sequenced at high depth (20×). The overall concordance rate was 98%, demonstrating that the imputed data were suitable for GWAS (Supplementary Table S1). The FCR values followed a normal distribution, as determined using SPSS 26.0 (Supplementary Figure S1 and Table S2). We conducted GWAS using the MLMA-LOCO algorithm in GCTA, which utilized a genetic relatedness matrix (GRM) and the “leave one chromosome out” (LOCO) approach to prevent proximal contamination. A total of 211 significant SNPs located on chromosomes 1, 2, 5, and 6 were identified using a threshold of *p* < 5E−08 (Figure 1).

### 3.2. Differential Gene Transcription in Pigs with High and Low Feed Efficiency

**Figure 2:**
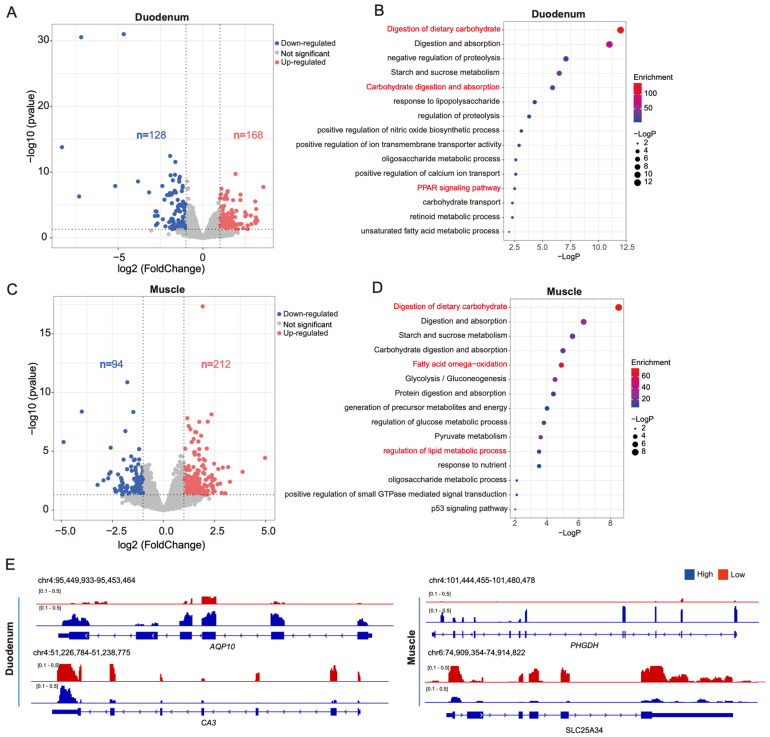
Analysis of differentially expressed genes in purebred large white boars with high and low FCR. (**A**,**C**) Volcano plot of differentially expressed genes in the duodenum (**A**) and muscle (**C**); *n* = 3, |log2(fold change)| ≥ 1 and *p* < 0.05. Blue dots indicate significantly downregulated genes, red dots indicate significantly upregulated genes, and gray dots indicate genes with no significant expression difference. (**B**,**D**) Functional enrichment analysis of differentially expressed genes in the duodenum (**B**) muscle tissue (**D**). (**E**) Transcriptional differences in the *AQP10*, *CA3*, *PHGDH*, and *SLC27A3* genes in the duodenum and muscle tissue between high- and low-FCR pigs.

To investigate the gene expression patterns in pigs with high and low FCR, we extracted high-quality RNA from three pigs with high FCR and three pigs with low FCR (Supplementary Table S3). We used this RNA to construct libraries and perform RNA-seq (Supplementary Figure S2 and Table S4). In a clustering heatmap, the duodenum and muscle tissues formed two distinct branches, indicating that there are significant differences in gene expression between the two tissues (Supplementary Figure S3). They also indicated that the sequencing depth was sufficient for the identification of DEGs (defined as |log_2_ fold change| in expression ≥ 1 and *p* < 0.05). There were 296 DEGs between the duodenum tissues of pigs with high and low FCR. In comparison with low-FCR pigs, high-FCR pigs had 168 upregulated DEGs and 128 downregulated DEGs (Figure 2A). Similarly, in muscle tissue, there were 306 DEGs, with 212 upregulated DEGs and 94 downregulated DEGs identified in high-FCR pigs compared with their low-FCE counterparts (Figure 2C).

Gene Ontology (GO) analysis revealed that DEGs in the duodenal tissue were primarily enriched in the biological process terms digestive absorption processes, carbohydrate metabolism, PPAR signaling pathway, and regulation of transporter proteins (Figure 2B), such as *AQP10* [18], which is associated with intestinal water and ion transport functions (Figure 2E). In muscle tissue, the main enriched biological processes terms were fatty acid oxidation, glycolysis, and lipid metabolism processes, such as *PHGDH* [19], which is associated with fatty acid oxidation metabolism (Figure 2E). These results indicate that the DEGs between high- and low-FCR pigs may regulate feed efficiency by participating in pathways regulating intestinal digestion and absorption, as well as energy metabolism.

### 3.3. Differences in Chromatin Accessibility between High- and Low-FCR Pigs

**Figure 3:**
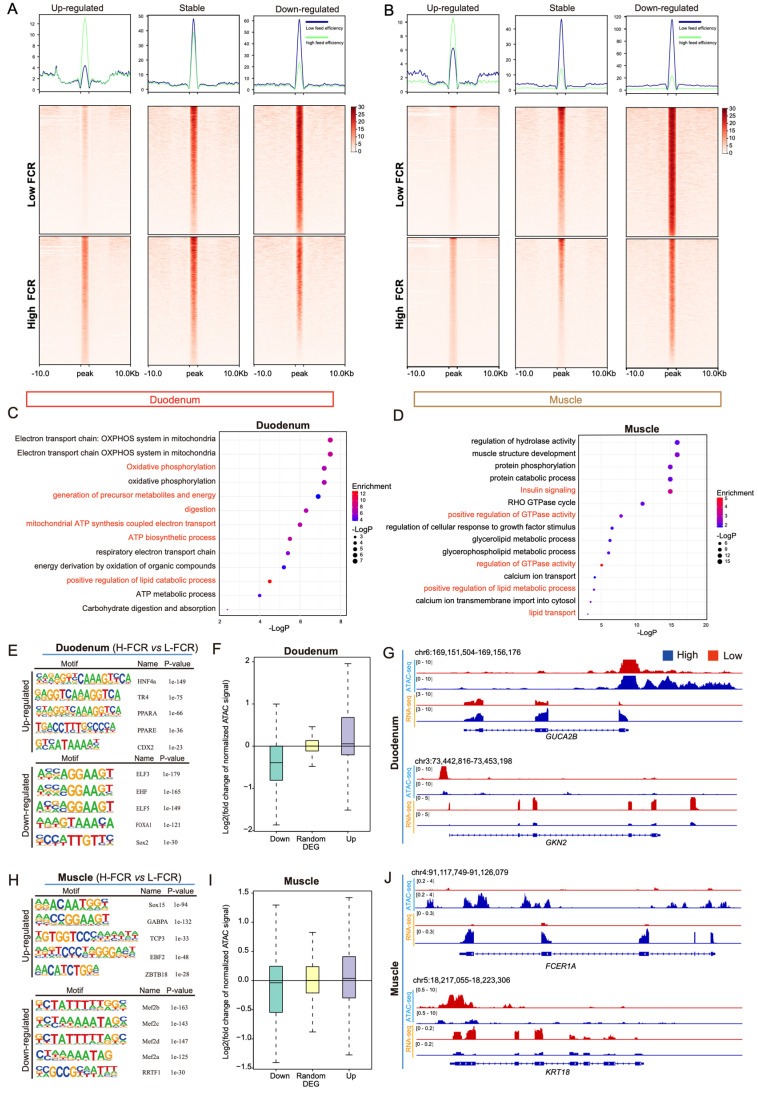
Analysis of differential chromatin accessibility between high- and low-FCR purebred large white boars. (**A**,**B**) Heatmaps showing differential ATAC-seq peaks in the duodenal and muscle tissues between high- and low-FCR pigs. (**C**,**D**) Results of functional enrichment analysis of genes associated with up- and downregulated ATAC-seq peaks. (**E**,**H**) Motifs associated with differential ATAC-seq peaks in duodenal (**E**) and muscle (**H**) tissues. (**F**,**I**) Box plots showing ATAC-seq signal intensities in the promoter regions of differentially expressed genes in the duodenum (**F**) and muscle (**H**). (**G**,**J**) Genomic snapshots of the promoters (**G**) and transcripts (**J**) of *GUCA2B*, *GKN2*, *FCER1A*, and *KRT18*.

We next conducted ATAC-seq on duodenal and muscle tissues from low- and high-FCR pigs to analyze differences in chromatin accessibility, which is associated with gene expression. The statistics of the sequencing data indicated that the ATAC-seq data were of high quality and had a sufficient sequencing depth, making them suitable for downstream analysis of differential chromatin accessibility between high- and low-FCR pigs (Supplementary Figure S4, Tables S5 and S6). We identified the OCRs in the high- and low-feed-efficiency groups and found that in comparison with low-FCR pigs, high-FCR pigs had 23,515 differential OCRs (10,270 upregulated peaks and 9302 downregulated peaks) in the duodenal tissue (Figure 3A) and 37,786 differential OCRs (24,775 upregulated peaks and 17,111 downregulated peaks) in the muscle tissue (Figure 3B). GO enrichment analysis of the genes associated with these differential OCRs revealed enrichment in pathways related to digestive absorption and lipid and carbohydrate metabolism activities in duodenal tissue (Figure 3C) and pathways related to triglyceride metabolism and energy metabolism in muscle tissue (Figure 3D).

We next explored the transcription factors with putative binding sites within these differential OCRs. Motif analysis revealed potential binding sites for transcription factors in the differential OCRs of duodenal and muscle tissues (Figure 3E). Notable transcription factors included HNF4a, TR4, PPARa, and PPARE in duodenal tissue and GABPA, TCP3, and ZBTB18 in muscle tissue. These transcription factors are predominantly involved in lipid and energy metabolism.

Chromatin accessibility can directly reflect the activity of regulatory elements [20]; therefore, we investigated the correlation between differences in chromatin accessibility within promoter regions and differences in the expression of genes between high- and low-FCR pigs. Chromatin accessibility was coordinated with changes in target gene expression, suggesting that the differences between high- and low-FCR pigs are mainly due to genes in pathways related to digestion, absorption, and energy metabolism. For instance, a gene associated with nutrient absorption and energy metabolism in the gut, *GUCA2B*, was highly expressed in the intestinal epithelial cells [21], and significant differences in chromatin accessibility and transcription levels of this gene were observed between the duodenal tissues of high- and low-FCR pigs (Figure 3F–J).

### 3.4. Integration of Multi-Omics Data for Identification of Key Functional Variants Underlying GWAS Signals

The main objective of this study was to identify genetic variations associated with feed efficiency traits through GWAS analysis. GWAS revealed 15 significant SNPs associated with FCR. Functional annotation of genes near these SNPs identified four significantly annotated SNPs within *PPP1R14C*, *MDGA2*, *TH*, and *KCNQ1*. An additional six significant SNPs fell within 0.8–9.7 kb from the nearest gene (Supplementary Table S7). Functional annotation information from epigenomic analysis was used to identify key functional variants affecting FCR. By combining RNA-seq, ATAC-seq, and GWAS data, DEGs, differential OCRs, and SNPs significantly associated with FCR in the duodenum and muscle of high- and low-FCR pigs were obtained, providing a data foundation for the functional annotation and mechanistic analysis of key variants associated with FCR.

**Figure 4:**
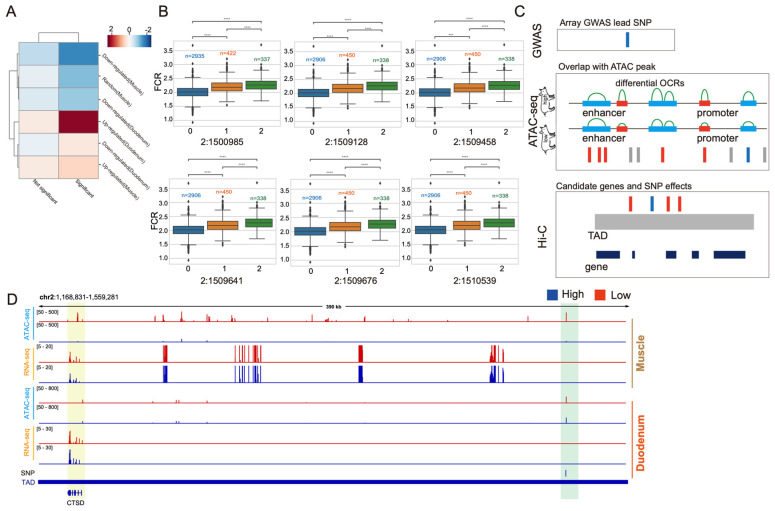
Integrated analysis of feed conversion rate and key functional variants. (**A**) GWAS signals within differential open chromatin regions. The *x*-axis represents GWAS significant SNPs (sig), non-significant SNPs (not sig) as a control (random 1000 SNPs across the genome), while the *y*-axis corresponds to ATAC-seq peak signals. (**B**) Phenotypes of individuals with different SNP genotypes. The six SNPs were selected from multi-omics analysis. On the *x*-axis, 0 represents homozygous in the reference genotype, 1 represents heterozygous, and 2 represents homozygous for the mutant allele. *** and **** indicate *p <* 0.001 and *p <* 0.0001, respectively (**C**) Strategy for identifying key functional variants and target genes. (**D**) Genomic snapshot of identified key functional variants and their target genes.

The impact of genetic variations is generally limited within topologically associated domain (TAD) regions, and cis-regulatory elements mainly interact within the same TAD region [22]. Therefore, we used TADs to locate target genes associated with SNPs. Toward this end, significant SNPs associated with feed efficiency were first obtained through GWAS analysis, and SNPs located within differential OCRs identified by ATAC-seq (Figure 4B) were retained. Next, we selected feed efficiency-related genes located within the TAD regions near the retained SNPs. RNA-seq data were then used to compare the transcript levels of these target genes in the duodenum and muscle tissues of high- and low-FCR pigs to identify candidate genes affecting feed efficiency (Figure 4D). Using this strategy, we identified six key functional variants associated with 12 known genes (Supplementary Table S8). The impact of the six SNPs (2:1500985, 2:1509128, 2:1510539, 2:1509641, 2:1509458, and 2:1509676) on FCR traits was then observed. The phenotype values for feed efficiency traits in individuals with homozygous variants (AA, GG, CC, TT, CC, GG) were consistently higher than in those with other genotypes (Figure 4C). These results indicate that the identified six SNP loci are significantly associated with feed efficiency traits. We next performed functional annotation of 12 known genes (*SYT8*, *TNNI2*, *ASCL2*, *MOB2*, *DUSP8*, *TH*, *LSP1*, *PRR33*, *TNNT3*, *IFITM10*, *CTSD*, and *INS*), retaining those related to FCR. For instance, five SNPs in the 2:1509128–1509676 region showed enrichment of ATAC-seq signals in the duodenum tissue of high-FCR pigs, with no signal enrichment observed in muscle tissue. When comparing the transcription levels of known genes within the same TAD region, we found that the *CTSD* gene exhibited high expression in the duodenum tissue of high-FCR pigs (Figure 4D).

## 4. Discussion

Feed efficiency is closely associated with digestion, the absorption of nutrients, and energy metabolism. The duodenum, an essential site for nutrient absorption in pigs, serves as one of the starting points for energy metabolism [23]. However, the scarcity of omics data resources for the pig duodenum has hindered elucidation of candidate genes and their causal variants. In this study, we conducted a GWAS analysis for the FCR trait and identified 15 significant SNPs. We performed functional annotation of these significant SNPs and found that four of them are located within genes, including 1:15865487, 2:1502986, and 2:1787269. PPP1R14C is a phosphatase reported to be a major regulator of glycogen synthase kinase 3β (GSK 3β), which is involved in glycogen synthesis and storage [24]. The expression of tyrosine hydroxylase (TH) in the gastrointestinal tract affects glucose homeostasis [25]. KCNQ1 is an ion channel protein that regulates gastric acid secretion, salt and glucose homeostasis, and cardiac rhythm [26]. Here, we identified that the PPP1R14C, TH, and KCNQ1 genes are associated with pathways related to glycogen metabolism and glucose homeostasis. These genes may influence feed efficiency in pigs by regulating energy metabolism. To further precisely identify key functional mutations and target genes associated with feed efficiency traits, we co-localized differential OCRs identified by ATAC-seq and significant SNPs identified by GWAS. We found that these loci were significantly enriched in upregulated OCRs in high-FCR pigs. We then identified a set of SNPs located within differential OCRs and found that all these SNPs clustered on chromosome 2. Observation of FCR in individuals with different SNP genotypes revealed that individuals that were homozygous for the mutant alleles had consistently higher feed efficiencies than those with other genotypes. This indicates a significant association between these SNPs and FCR. We investigated the ATAC-seq signals for six SNP loci and found that the signals in the region 2:1509128–1509676, where five SNPs are located, were significantly enriched in the duodenum tissue of high-FCR pigs, while no significant enrichment was observed in muscle tissue. By comparing the transcription levels of known genes within the same TAD region using RNA-seq data, we found that the *CTSD* gene was highly expressed in the duodenum of high-FCR pigs. OCRs identified by ATAC-seq are often associated with regulatory elements such as promoters and enhancers, and interactions within a TAD are stronger than those outside the TAD [27]. Thus, these SNPs may regulate the expression of other genes within the same TAD by affecting regulatory elements such as promoters or enhancers. Therefore, we speculate that the five SNPs in the region 2:1509128–1509676 may be located within regulatory elements (promoter or enhancer) at the distal end of the *CTSD* gene, promoting its expression in the duodenum tissue of high-feed efficiency pigs. CTSD is an aspartic protease associated with lysosomes, regulating the breakdown of lipids and playing a crucial role in lipid synthesis and metabolism [28]. In rodents, CTSD has been linked to insulin sensitivity [29], and insulin expression can promote glucose uptake, glycolysis, and regulate lipid metabolism [30]. A significant association has been observed between a CTSD mutation (g. 70 G>A) and feed efficiency traits in Italian duroc pigs [31].

Nutrients are primarily absorbed through the intestinal epithelium of the duodenum and enter the bloodstream, making them available to other parts of the body. Increasing the efficiency of absorption in the duodenum can promote pig growth and weight gain, ultimately enhancing feed efficiency. For example, one study found that an increase in the expression levels of glucose transporter genes in the pig small intestine can elevate the absorption rate of glucose, thereby improving feed utilization efficiency [32]. Another study found that there are individual differences in the expression levels of amino acid and fatty acid metabolism genes in the small intestine. Upregulation of the expression of these genes can significantly enhance the efficiency of amino acid and fatty acid utilization in the small intestine, thereby increasing feed conversion efficiency [33,34]. We chose the duodenum as the research subject because it is the initial segment of the small intestine, where many critical digestive and absorptive processes begin. The duodenal mucosa is rich in glandular secretory cells, playing a vital role in neutralizing chyme and enzymatic digestion. Numerous studies have found that gene mutations can lead to metabolic abnormalities in the duodenum, directly affecting nutrient absorption and energy conversion efficiency in the body [35,36]. However, nutrient absorption occurs not only in the duodenum but also extensively in the jejunum and ileum [8,37]. Therefore, future research should consider conducting similar gene identification studies in other parts of the small intestine, such as the jejunum and ileum, to eliminate potential regional differences and gain a comprehensive understanding of nutrient absorption in the small intestine.

In summary, by integrating multi-omics data, we identified six SNP loci significantly associated with feed efficiency traits. Among these, five (2:1500985, 2:1509128, 2:1510539, 2:1509641, 2:1509458, and 2:1509676) may be located within the promoter or an enhancer of the *CTSD* gene, promoting the expression of *CTSD* in the duodenum tissue of high-feed-efficiency pigs. Our findings suggest that *CTSD* is related to pig feed efficiency.

## Data Availability

The omics datasets generated in the present study are available at the Gene Expression Omnibus (www.ncbi.nlm.nih.gov/geo/ accessed on 17 June 2024) under the accession number GSE270124.

## Supplementary Materials

The following supporting information can be downloaded at https://www.mdpi.com/article/10.3390/genes15080980/s1. Figure S1: Distribution and Q-Q plot of feed conversion ratio (FCR) in 3688 boars. Figure S2: Genomic RNA samples’ 1.5% agarose gel electropherogram. Figure S3: Heatmap of the correlations among the RNA-seq data from all samples. Figure S4: Distributions of the sizes of DNA fragments from ATAC-seq libraries constructed for duodenal and muscle tissues of low- and high-feed-efficiency pigs. Figure S5: ATAC-seq library insert lengths, tissue correlations, and TSS enrichment. Figure S6: Comparative Analysis of Pig Body Weight Across Ages: Identifying Abnormal and Normal Growth Patterns. Table S1: Genotype filling accuracy. Table S2: GWAS phenotype data statistics. Table S3: Statistics of individuals in extreme groups. Table S4: Summary of RNA-seq data from high- and low-feed-efficiency pigs. Table S5: Summary of ATAC-seq data from high- and low-feed-efficiency pigs. Table S6: Quality control values for ATAC-seq data. Table S7: Functional annotation of SNPs significantly associated with FCR. Table S8: Key functional variants retained by integromics screening.
