# Supplementary material for: Integrating Multi-Omics Data to Identify Key Functional Variants Affecting Feed Efficiency in Large White Boars
Source: Genes (Basel). 2024 Jul 25;15(8):980. doi: 10.3390/genes15080980 (PMC11353296; doi:10.3390/genes15080980)
Supplement: Supplementary file 1 [file genes-15-00980-s001.zip › genes-3094668-supplementary.pdf]

Supplementary material

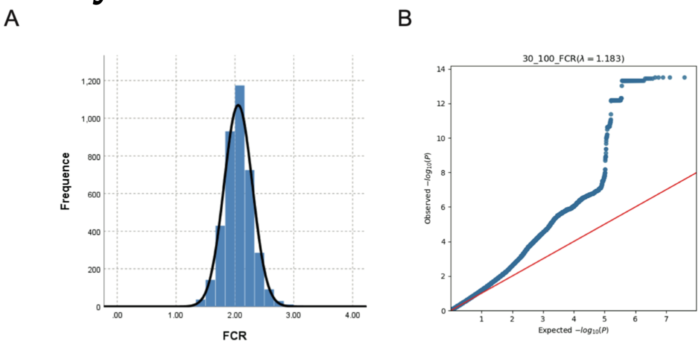

Supplementary Figure S1 (A) Plot of FCR values from 484 boars. The data follow a normal distribution. (B) Q-Q plot for FCR.

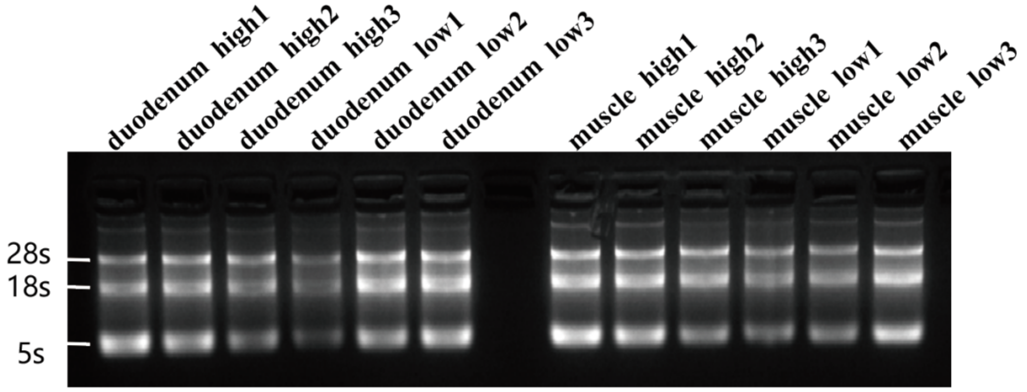

Supplementary Figure S2 Genomic RNA samples 1.5% agarose gel electropherogram.

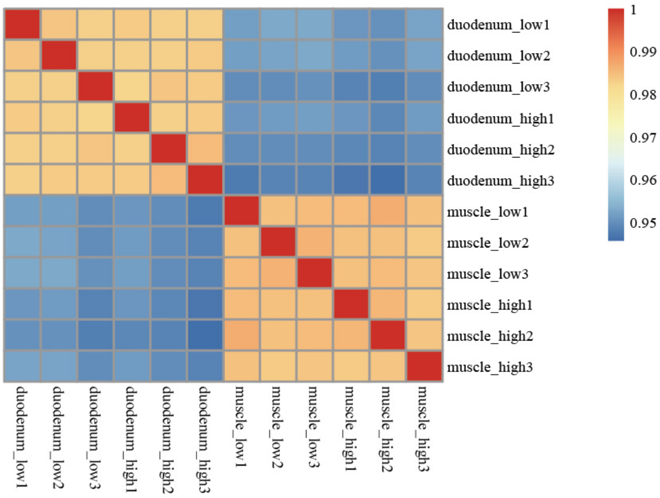

Supplementary Figure S3 Heatmap of the correlation between the RNA-seq data from all samples.

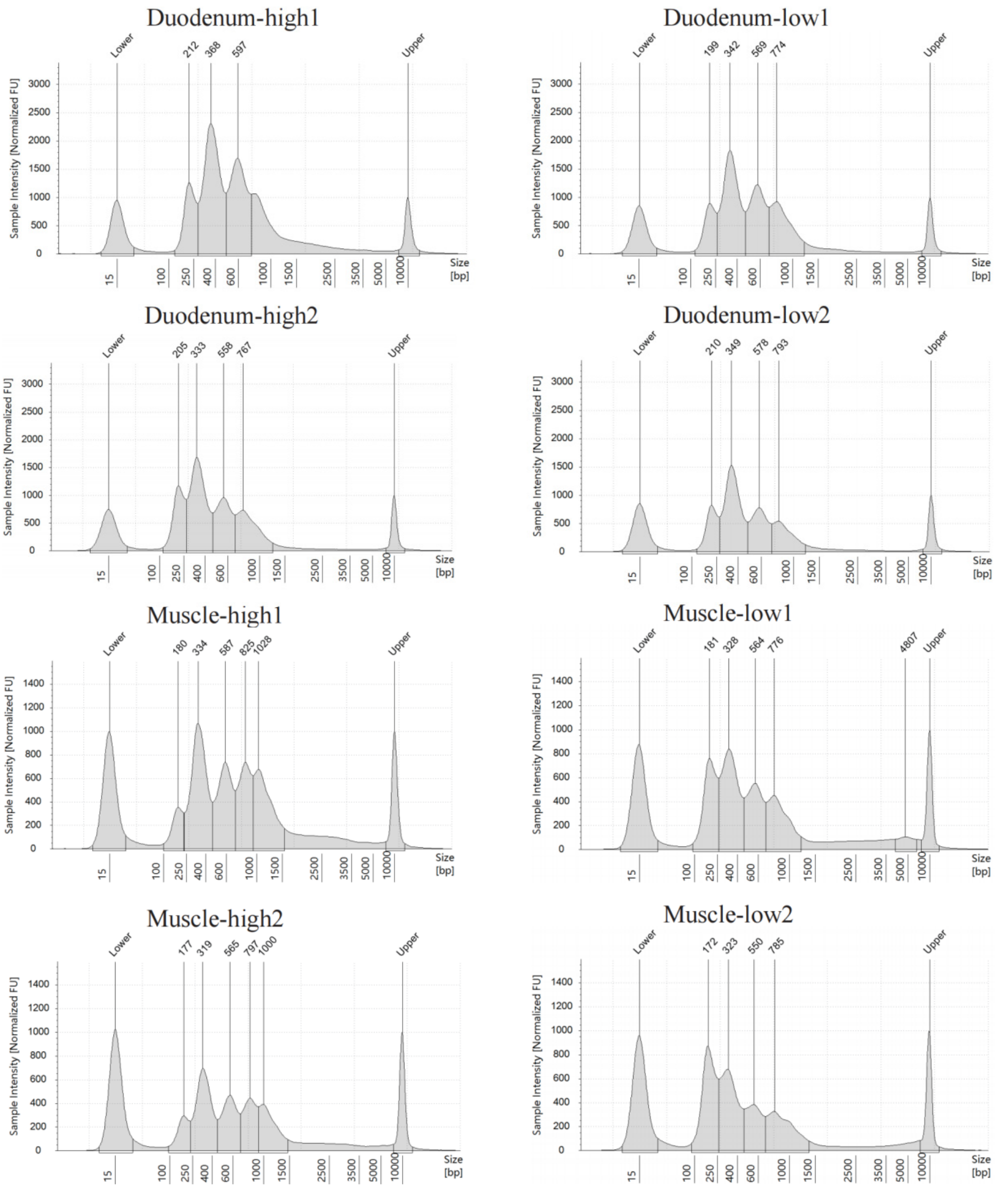

Supplementary Figure S4 Distributions of the sizes of DNA fragments from ATAC-seq libraries constructed for duodenal and muscle tissues of low- and high-feed efficiency pigs.

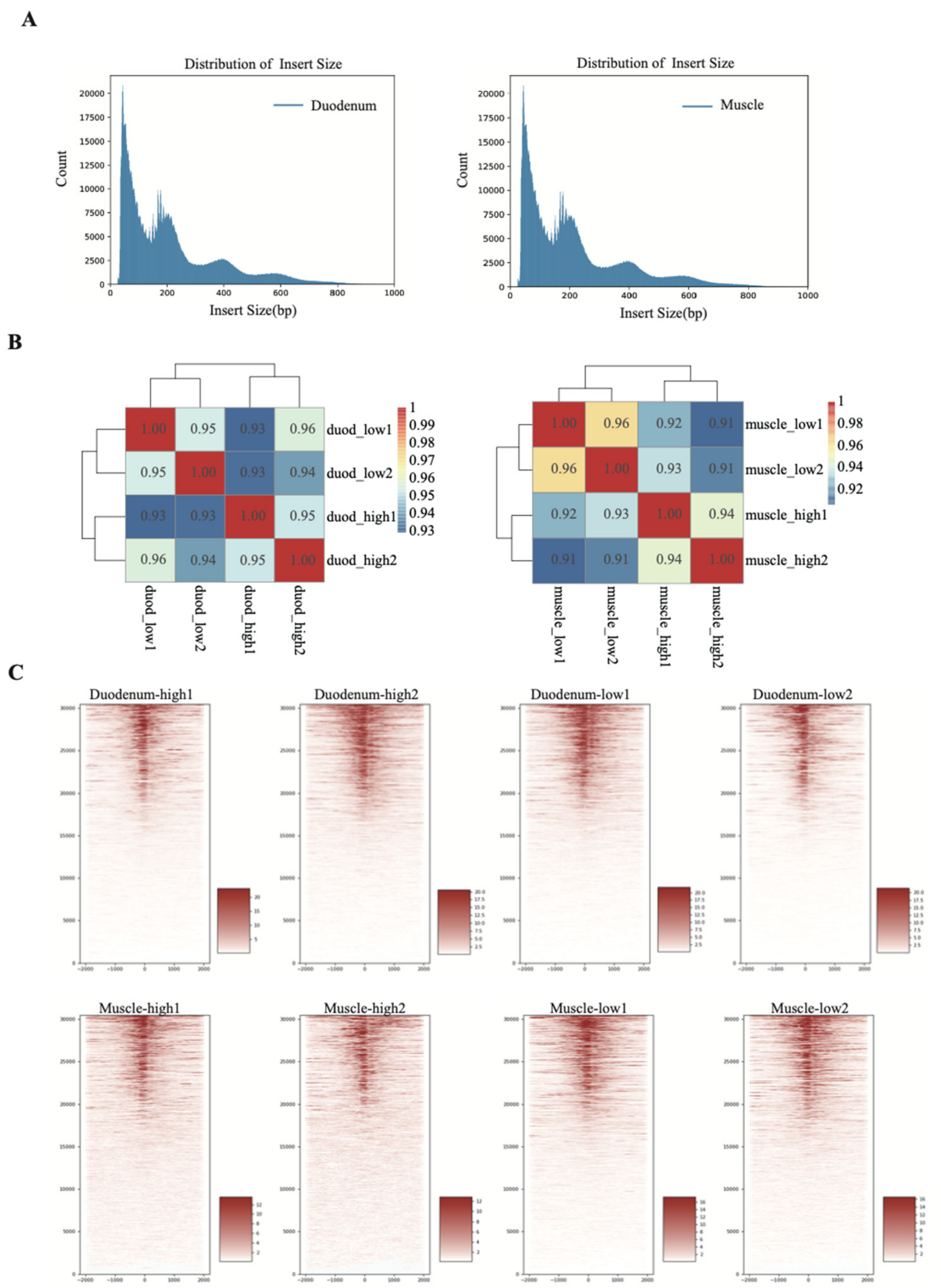

Supplementary Figure S5 (A) Length distributions of ATAC-seq library inserts for the duodenum (left) and muscle (right). (B) Heatmaps showing the correlations between ATAC-seq data for duodenal and muscle tissues. (C) TSS enrichment heat map of ATAC-seq data.

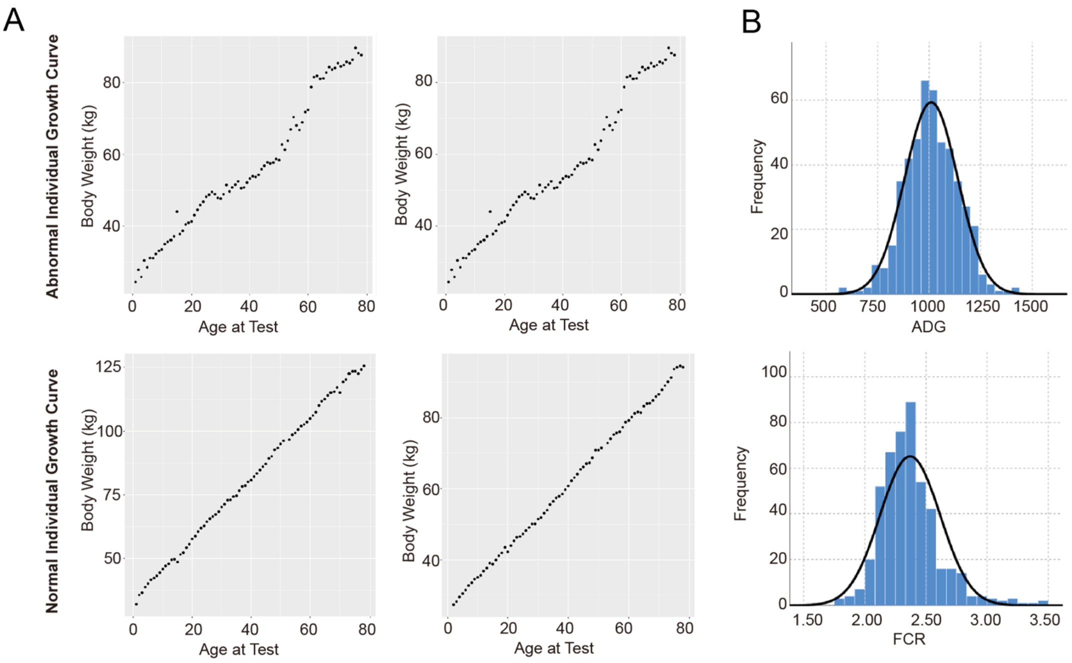

Supplementary Figure S6 (A) Serial pig body weight (kg) plotted against age (day). Dots in plots in the top row represent abnormal pig body weight against age; dots in plots in the bottom row represent normal pig body weight, with age (days) on the x-axis. (B) Phenotype distribution map.

Supplementary Table S1 Genotype filling accuracy

Individual identification	Genotype consistency	Sequencing depth	Coverage 1X	Coverage 5X
YYOOOST220015504	0.985009291	23.7754X	98.12%	96.38%
YYOOOST220035001	0.984665261	27.359X	98.16%	96.78%
YYOOOST220037202	0.985389367	28.1842X	98.15%	96.99%
YYOOOST220055204	0.981174891	16.5572X	97.79%	92.03%

Supplementary Table S2 GWAS phenotype data statistics

Trait	Number	Min	Max	Mean	SD
FCR	3866	1.52	3.71	2.06	0.24

Supplementary Table S3 Statistics of individuals in extreme groups

Individual	Groups	FCR	Age(d)	Gender	Weight (kg)
FCR-H1	FCR-H	3.43	180	Boars	99.5
FCR-H2	FCR-H	3.42	180	Boars	101.5
FCR-H3	FCR-H	3.37	180	Boars	97.6
FCR-L1	FCR-L	1.82	180	Boars	110.5
FCR-L2	FCR-L	1.79	180	Boars	108.5
FCR-L3	FCR-L	1.77	180	Boars	105.5

Supplementary Table S4 Summary of RNA-seq data from high- and low-feed efficiency pigs

Sample	Raw reads	Raw bases (G)	Clean reads	Mapping (%)
Duodenum-FCR-H1	38735007	11.62	38278579	85.06
Duodenum-FCR-H2	32597117	9.78	32331541	83.12
Duodenum-FCR-H3	38795714	11.64	38503273	85.48
Duodenum-FCR-L1	32594531	9.78	32514768	83.42
Duodenum-FCR-L2	33467946	10.04	33237965	84.79
Duodenum-FCR-L3	33495905	10.05	33361326	79.50
Muscle-FCR-H1	42765941	12.83	42436202	91.51
Muscle-FCR-H2	46459005	13.94	46104937	91.64
Muscle-FCR-H3	47970760	14.39	47414865	90.88
Muscle-FCR-L1	37347196	11.20	37038379	91.38
Muscle-FCR-L2	39022113	11.71	38772541	90.12
Muscle-FCR-L3	34224867	10.27	34056939	88.88

Supplementary Table S5 Summary of ATAC-seq data from high- and low-feed efficiency pigs

Sample	Raw reads	Raw bases (G)	Clean reads	Mapped (%)
Duodenum-FCR-H1	396159016	45.32	396066470	88.70
Duodenum-FCR-H2	295635786	48.41	295554740	93.80
Duodenum-FCR-L1	304949624	51.48	304852854	94.10
Duodenum-FCR-L2	296427996	54.71	296358310	90.90
Muscle-FCR-H1	302164422	45.32	302085018	93.00
Muscle-FCR-H2	322741820	48.41	322644262	93.00
Muscle-FCR-L1	343197174	51.48	343018674	94.00
Muscle-FCR-L2	364713114	54.71	364566180	93.50

Supplementary Table S6 Comparison of ATAC-seq data from high- and low-feed efficiency pigs on a reference genome groups

Sample	NSC	RSC	FRiP	NRF
Duod-FCR-H1	1.362821	2.240229	0.25360968	0.808573
Duod-FCR-H2	1.334187	2.359746	0.280859647	0.802661
Duod-FCR-L1	1.410988	2.353384	0.304524411	0.822301
Duod-FCR-L2	1.79844	1.993465	0.356528252	0.788732
Muscle-FCR-H1	1.122996	2.482323	0.152923195	0.842437
Muscle-FCR-H2	1.093728	2.485174	0.129725683	0.726569
Muscle-FCR-L1	1.366419	2.303349	0.290910396	0.839881
Muscle-FCR-L2	1.24408	2.409691	0.241519379	0.842289

NSC: comparison of the correlation of positive and negative strand reads per chromosome; RSC: comparison of NSC values in the samples with those in the corresponding input samples; FRiP: ratio of the number of reads covering peaks to the total number of reads; NRF: ratio of the number of non-redundant reads to the total number of reads.

Supplementary Table S7 Functional annotation of SNPs significantly associated with FCR

Significant SNP	Chr	Position	*p*-Value	Nearest Gnen	Distance（bp）
1:15865487	1	15865487	1.73E-08	PPP1R14C	intron
1:176830754	1	176830754	2.57E-08	MDGA2	intron
1:179235855	1	179235855	3.90E-08	ENSSSCG00000046948	11891
1:179351187	1	179351187	1.28E-09	ENSSSCG00000046948	97645
2:1490739	2	1490739	3.14E-14	ENSSSCG00000035293	intron
2:1491672	2	1491672	1.20E-09	ENSSSCG00000035293	intron
2:1502986	2	1502986	3.83E-08	TH	intron
2:1787269	2	1787269	1.54E-08	KCNQ1	intron
2:3753565	2	3753565	1.25E-08	ENSSSCG00000046960	112109
5:21077603	5	21077603	2.83E-08	ENSSSCG00000031722	intron
6:56577794	6	56577794	3.02E-08	ENSSSCG00000038825	intron
6:57297029	6	57297029	2.98E-08	ENSSSCG00000045482	intron
6:57379002	6	57379002	4.55E-08	ENSSSCG00000045482	7175
6:57483029	6	57483029	1.74E-08	ENSSSCG00000033784	3244
6:58462356	6	58462356	2.07E-09	ENSSSCG00000022906	892

Supplementary Table S8 Key functional variants retained by integromics screening

SNP ID	Chr	Position	*p*-Value
2:1500985	2	1500985	4.85E-14
2:1509128	2	1509128	6.61E-13
2:1510539	2	1510539	6.61E-13
2:1509641	2	1509641	6.61E-13
2:1509458	2	1509458	6.61E-13
2:1509676	2	1509676	6.61E-13
